# Supplementary material for: SRPX Emerges as a Potential Tumor Marker in the Extracellular Vesicles of Glioblastoma
Source: Cancers (Basel). 2022 Apr 14;14(8):1984. doi: 10.3390/cancers14081984 (PMC9028996; doi:10.3390/cancers14081984)
Supplement: Supplementary file 1 [file cancers-14-01984-s001.zip › cancers-1596864-supplementary.pdf]

Supplementary Material: SRPX Emerges as a Potential Tumor Marker in the Extracellular Vesicles of Glioblastoma

Elisabet Ampudia-Mesias, Samia El-Hadad, Charles Scott Cameron, Adelheid Wöhrer, Thomas Ströbel, Nurten Saydam and Okay Saydam

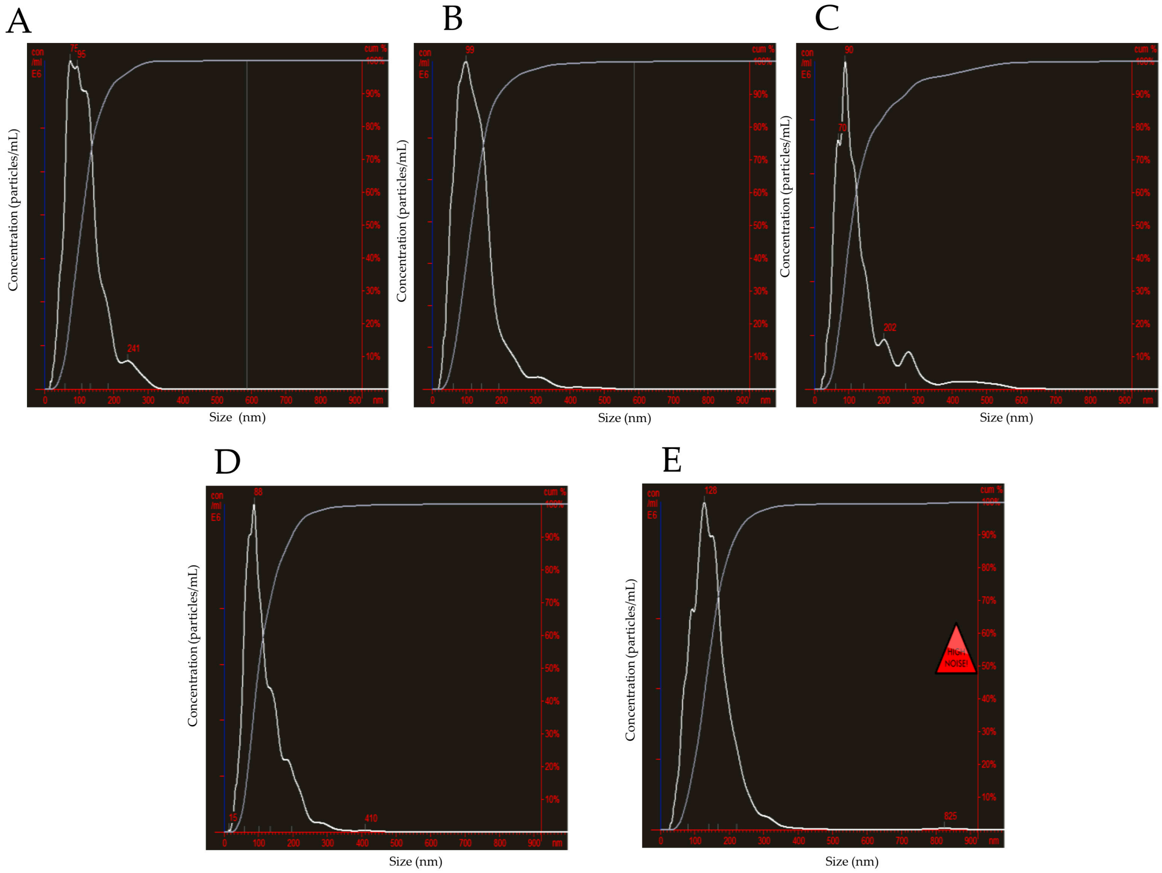

Figure S1. Nanoparticle Tracking Analysis of EVs shows that glioblastoma EVs display similar size. Representative nanoparticle tracker analysis of histograms and average diameter size of EVs are shown in (**A**) Human primary astrocytes 95 nm, (**B**–**E**) primary glioblastoma cell lines: 99 nm, 90 nm, 88 nm, 128 nm (IN-GB-9, IN-GB-11, IN-GB-28, IN-GB-29 respectively).

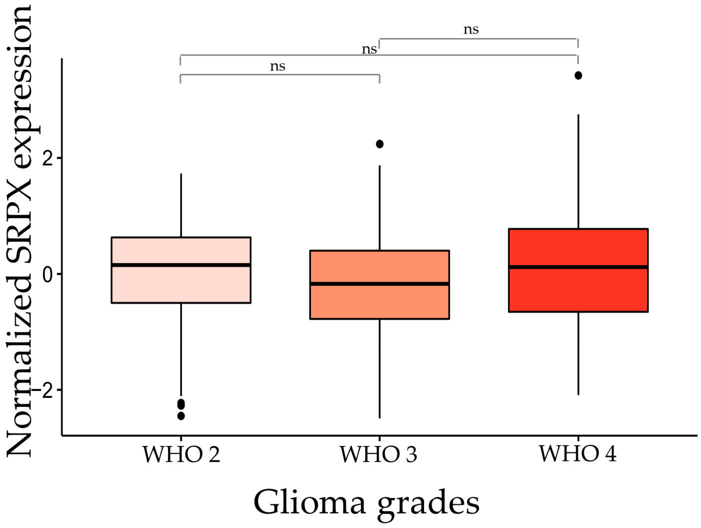

Figure 2. SRPX expression in gliomas. Normalized SRPX expression in glioma tumor grades in mRNAseq_325 dataset from CGGA database. Kruskal-Wallis was used to compare SRPX mRNA expression among more than two groups and Dunn's test was used to adjust multiple comparisons. Ns: not significant.

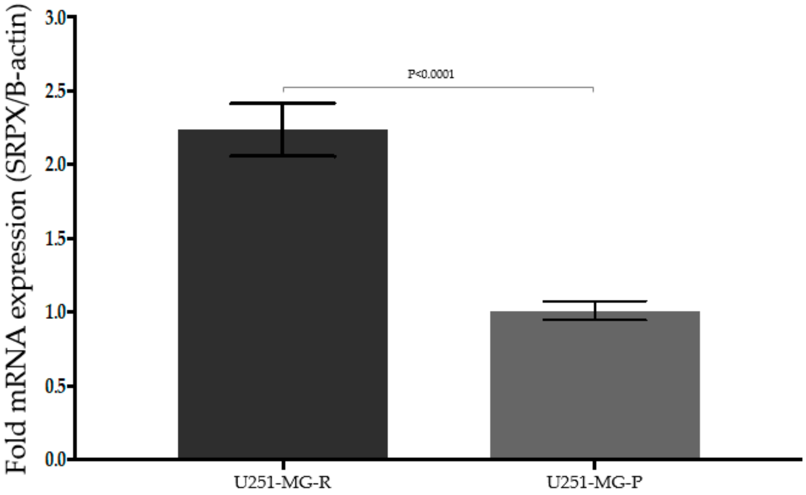

Figure S3. Endogenous transcript levels of SRPX in U251-MG-P and U251-MG-R cells. U251-MG-P and U251-MG-R cells were harvested, and mRNA was isolated to determine the SRPX transcript levels. Relative expression analysis was performed using the ΔΔCq method and gene expression was normalized to hB-actin. Error bars indicate means ± SEM and *p*-values indicate significance between each cell line (Bonferroni or Dunnett multiple comparisons test).

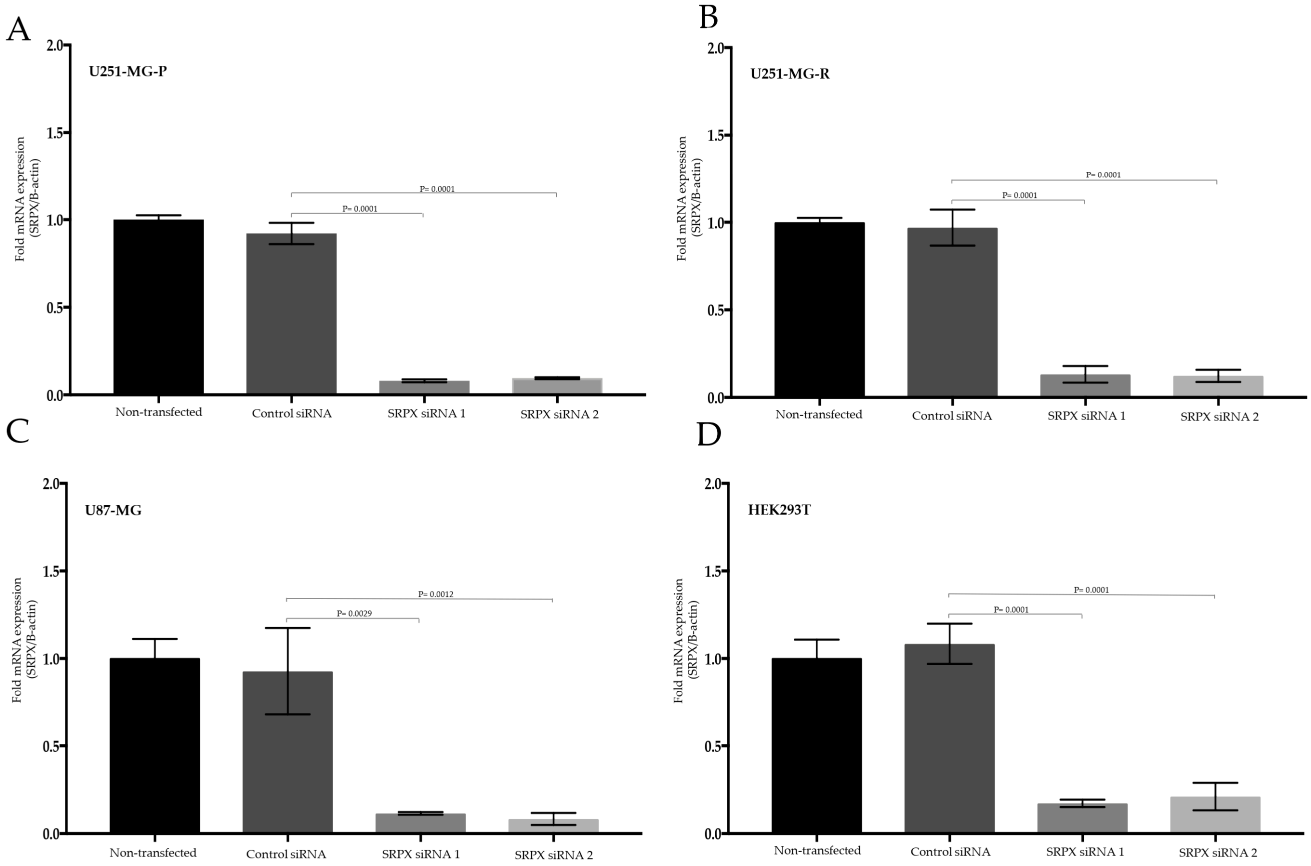

Figure 4. SRPX silencing in glioblastoma and HEK293T cells. (**A**) U251-MG-P or (**B**) U251-MG-R or (**C**) U87-MG, or (**D**) HEK293T, were transfected with SRPX siRNA or control siRNA (final concentration: 100 nM) or left non-transfected. Silencing of SRPX was confirmed by real-time PCR analysis. Error bars indicate means ± SEM and p-values indicate significance between indicated treatment groups (Bonferroni or Dunnett multiple comparisons test).

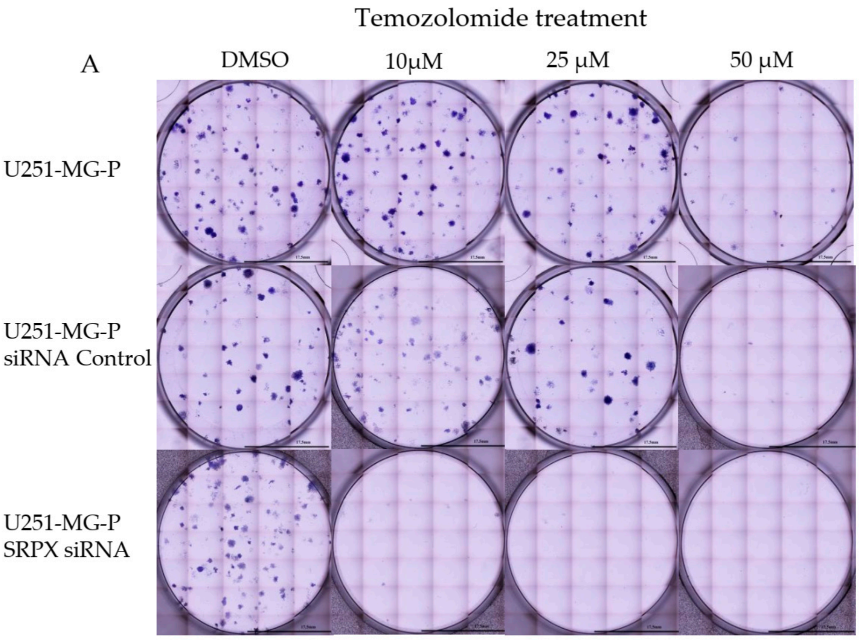

B
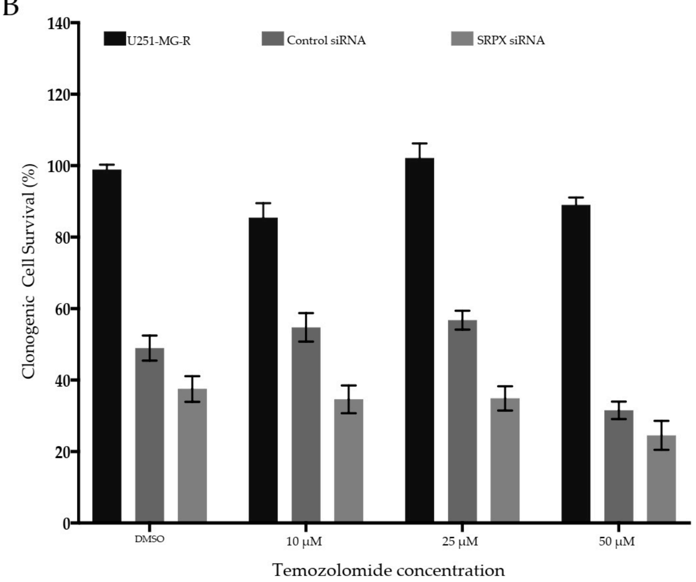

Figure S5. Silencing SRPX sensitizes U251-MG-P to TMZ. (**A**) Representative images of colony formation assay showing U251-MG-P colony survival after being exposed to TMZ. (**B**) Clonogenic cell survival percentage of U251-MG-R cells. Error bars indicate means ± SEM.

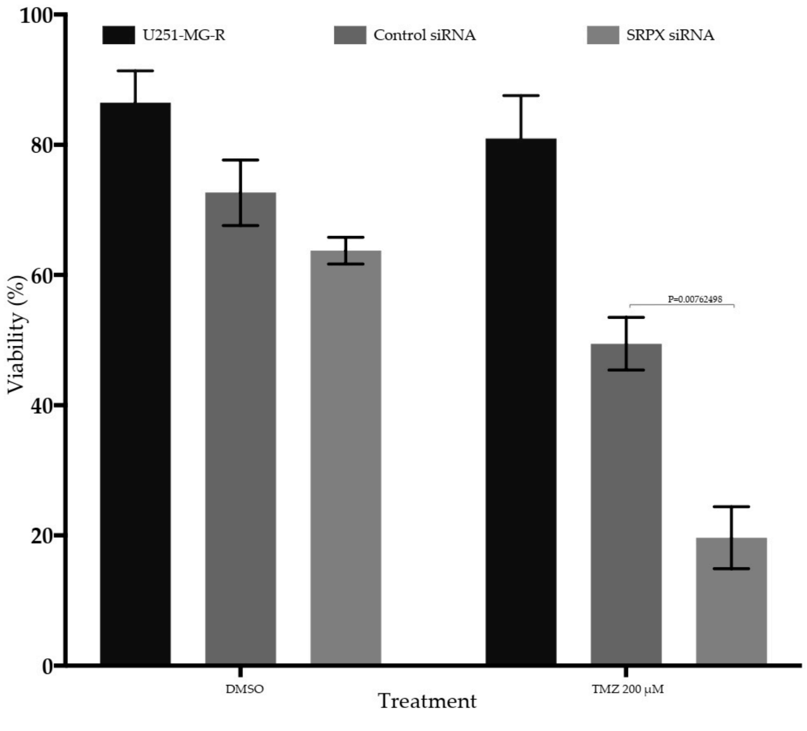

Figure S6. SRPX silencing decreases cell viability of U251-MG-R Cells. Percentage of viability of U251-MG-R cells after being exposed to TMZ. U251-MG-R cells were transfected with SRPX siRNA or control siRNA or left non-transfected. 24 h later, cells were exposed to 200 μM of TMZ, and after 48 h, cells were stained with trypan blue and counted by using a Neubauer cell counting chamber. Error bars indicate means ± SEM and p-values indicate significance compared to cells treated with control siRNA (Bonferroni or Dunnett multiple comparisons test).

Table S1. The Summary Statistics of Found Protein per each Cell.

Sample ID	Protein count	ID count	Spectra count	Fraction of IDs
HPA CL	184	908	10996	0.082575

HPA EV	67	578	21505	0.026877
IN-GB-9 CL	253	1634	23351	0.069976
IN-GB-9 EV	20	140	19965	0.007012
IN-GB-11 CL	311	2283	27045	0.084415
IN-GB-11 EV	15	54	19188	0.002814
IN-GB-28 CL	264	1884	23610	0.079797
IN-GB-28 EV	52	529	22425	0.02359
IN-GB-29 CL	262	1859	26338	0.070582
IN-GB-29 EV	25	169	20791	0.008129

Table S2. Mutation Status of IDH1 and IDH2 Genes, TERT Promoter and MGMT Promoter Methylation.

Sample-ID	WHO Grade	IDH1	IDH2	TERT Promoter	MGMT Promoter	Treatments
*Sample 1	2	R132H	WT	WT	Methylated	
Sample 2	2	WT	WT	WT	Unmethylated	
Sample 3	2	WT	WT	WT	Unmethylated	
Sample 4	3	WT	WT	C228T	Unmethylated	
*Sample 5	3	R132H	WT	WT	Methylated	
Sample 6	3	R132H	WT	WT	Unmethylated	
Sample 7	4	WT	WT	C228T	Methylated	TMZ+IR
Sample 8	4	WT	WT	C228T	Unmethylated	TMZ+IR
*Sample 9	4	WT	WT	WT	Methylated	Only IR
Recurrent of:						
Sample 7	4	WT	WT	C228T	Methylated	TMZ+IR
Sample 8	4	WT	WT	WT	Unmethylated	TMZ+IR
*Sample 9	4	WT	WT	WT	Unmethylated	Only IR

*Samples used in figure 2B; WT: Wild Type; R132H: Mutation in IDH1 gene; C228T: Mutation in TERT promoter; IR: Ionizing radiotherapy; TMZ: Temozolomide.

Table S3. Primer Sequences.

Gene	Primer set	Orientation	Sequence (5' - 3')	Associated Figure
SRPX	Set 1	Forward	CTGCACTTCTGGATCAGTTT	Figure 4A–B
SRPX	Set 1	Reverse	AGGCATAATCTTTGCTCCTATC	Figure 4A–B
GAPDH	Set 1	Forward	TTGATGGTACATGACAAGGTG	Figure 4A–B
GAPDH	Set 1	Reverse	AATTTGGCTACAGCAACAGGG	Figure 4A–B
ACTB	Set 1	Forward	CACTGTGCCCATCTACGAGG	Figure 4A–B
ACTB	Set 1	Reverse	AGGTAGTCAGTCAGGTCCCG	Figure 4A–B
